# Identification of cytotoxic agents disrupting synovial sarcoma oncoprotein interactions by proximity ligation assay

**DOI:** 10.18632/oncotarget.8882

**Published:** 2016-04-21

**Authors:** Aimée N. Laporte, Jennifer X. Ji, Limin Ma, Torsten O. Nielsen, Bertha A. Brodin

**Affiliations:** ^1^ Department of Pathology and Laboratory Medicine, Vancouver Coastal Health Research Institute and Faculty of Medicine, University of British Columbia, Vancouver, BC, Canada; ^2^ Centre for Translational and Applied Genomics, British Columbia Cancer Agency, Vancouver, BC, Canada; ^3^ Department of Oncology and Pathology, Karolinska Institutet, Stockholm, Sweden

**Keywords:** synovial sarcoma, proximity ligation assay, drug screening, HDAC inhibitors, protein-protein association

## Abstract

Conventional cytotoxic therapies for synovial sarcoma provide limited benefit. Drugs specifically targeting the product of its driver translocation are currently unavailable, in part because the SS18-SSX oncoprotein functions via aberrant interactions within multiprotein complexes. Proximity ligation assay is a recently-developed method that assesses protein-protein interactions *in situ*. Here we report use of the proximity ligation assay to confirm the oncogenic association of SS18-SSX with its co-factor TLE1 in multiple human synovial sarcoma cell lines and in surgically-excised human tumor tissue. SS18-SSX/TLE1 interactions are disrupted by class I HDAC inhibitors and novel small molecule inhibitors. This assay can be applied in a high-throughput format for drug discovery in fusion-oncoprotein associated cancers where key effector partners are known.

## INTRODUCTION

The chromosomal translocation t(X;18)(p11.2;q11.2) is the main cytogenetic event in synovial sarcoma and results in the fusion of the BAF complex member *SS18* with one of three highly homologous transcriptional repressor genes, *SSX1, SSX2* or *SSX4* [1, 2]. The resulting *SS18-SSX* fusion oncogene can be detected in nearly all synovial sarcomas and is used clinically to confirm the diagnosis. Importantly, the expression of *SS18-SSX* is necessary and sufficient for the development of synovial sarcoma, as demonstrated in transgenic mice wherein the conditional expression of *SS18-SSX* in early myoblasts leads to tumor development without the requirement of any cooperative transgenic changes [3].

The SS18-SSX fusion protein has been proposed to displace native SS18, leading to aberrant SWI/SNF-mediated gene transcription [4]. The fusion of SSX to SS18 also recruits interacting proteins involved in epigenetic regulation, including transducin-like enhancer of split 1 (TLE1), activating transcription factor 2 (ATF2), members of the polycomb group and histone deacetylases (HDAC) [5, 6]. Together, this is thought to bring about the abnormal transcriptional pattern that drives malignant transformation in synovial sarcoma [4, 5].

Polycomb recruitment by TLE1 and the fusion oncoprotein triggers the repression of genes targeted by the SS18-SSX/ATF2/SWI-SNF core through trimethylation of histone H3 at lysine 27 [5]. We have previously shown that the association of TLE1 with the SSX domain of the fusion oncoprotein results in the repression of ATF2 target genes, including early growth response-1 (*EGR1*), a key positive regulator of the PTEN tumor suppressor [5, 7]. This permits up-regulation of PI3K/AKT/mTOR signalling, resulting in a proliferative, anti-apoptotic phenotype [8]. When this association is disrupted by specific knockdown of TLE1, ATF2 or SS18-SSX, synovial sarcoma cell lines are observed to undergo apoptosis, indicating this complex association is important for tumor cell survival [5].

The SS18-SSX/TLE1/ATF2 protein complex has been shown to be disrupted following treatment with HDAC inhibitors [5], providing an explanation for the sensitivity of synovial sarcoma cells to HDAC inhibition [9]. Based on the finding that the SS18-SSX fusion oncoprotein is unique to synovial sarcoma and is necessary and sufficient for tumor initiation [10], small molecules able to disrupt the SS18-SSX protein complex may have selective anti-tumor activity in synovial sarcoma. As SSX is not expressed in somatic tissues, an attractive drug target in this system is the SSX/TLE1 interface.

In the present investigation, a proximity ligation assay (PLA) was used to screen for drugs that disrupt the interaction between SS18-SSX and TLE1. The method was developed to visualize protein associations and modifications *in situ* with very high specificity, at interaction distances within 30 nm (Olink Bioscience) [11, 12]. This assay methodology allows for the direct identification of proteins in such close proximity by utilizing protein-specific antibodies conjugated with oligonucleotides that are ligated and amplified using fluorophore-labelled primer sequences [13]. The resulting fluorescent signal can be detected by fluorescent microscopy. PLA has been used to detect protein complexes and post-translational modifications *in situ*, as well as for high-throughput screening practices, demonstrating potential for use in pre-clinical drug screening models [14-17]. PLA technology has further been used in excised tissues from *in vivo* studies to monitor disease state and therapy responses [18-20].

In this study, we apply the proximity ligation assay to show that the interaction of SS18-SSX with TLE1 is detectable only in synovial sarcoma, confirm that this interaction is disrupted by HDAC inhibitors, and identify novel molecules capable of disrupting this interaction using high-throughput drug screens. This work instantiates the value of the proximity ligation technique in uncovering compounds that disrupt oncogenic protein associations, applicable to important oncogenic mechanisms among the growing collection of neoplasms driven by translocation-associated fusion oncoproteins.

## RESULTS

### The proximity ligation assay detects SS18-SSX/TLE1 co-localization *in situ*

**Figure 1:**
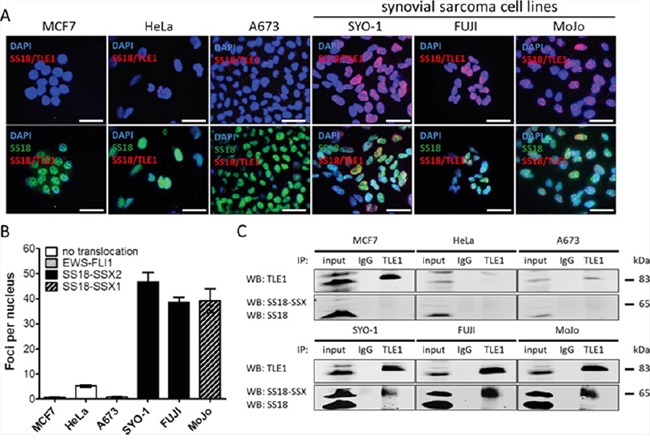
The proximity ligation assay demonstrates SS18-SSX/TLE1 co-localization selectively in synovial sarcoma cell lines SS18-SSX positive cell lines (SYO-1, FUJI, MoJo) demonstrate SS18-SSX/TLE1 proximity by visualization of fluorescent nuclear foci while control cell lines (MCF7, HeLa, A673) showed little nuclear staining **A.** The intensity of the nuclear signal was quantified and shown to be significantly greater in SS18-SSX confirmed-positive cell lines **B.** The SS18-SSX/TLE1 protein-protein interaction was confirmed to be specific for synovial sarcoma cell lines by co-immunoprecipitation **C.** Scale bars represent 20 μm. Error bars represent standard error of mean from three independent studies.

**Figure 2:**
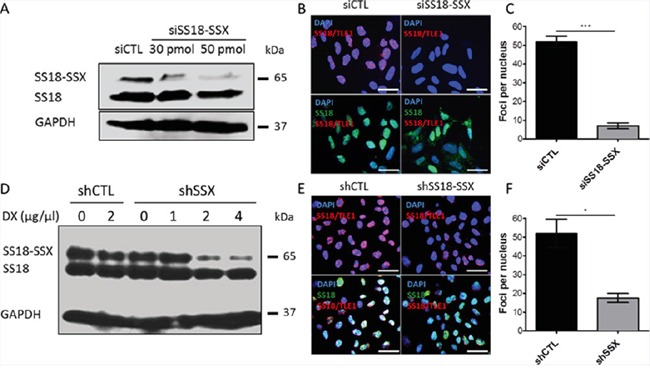
Knockdown of SS18-SSX results in a loss of SS18-SSX/TLE1 co-localization and a significant decrease in PLA nuclear signals 50 pmol of siRNA was used to knockdown SS18-SSX in SYO-1 cells, as demonstrated by a loss of protein expression. Protein levels **A.** and PLA signals **B, C.** were analyzed 48 hours post siRNA treatment. CME-1 cells were stably transfected with a doxycycline-inducible shRNA against SS18-SSX. Protein level **D.** and PLA signals **E, F.** were assessed at 24 hours post 4 μg/μL doxycycline (DX) treatment. Scale bars represent 20 μm. Statistical significance compared to RNA interference controls was determined by Student t test: * denotes *p* < 0.05; ** denotes *p* < 0.01. Error bars represent standard error of mean from three images.

In order to visualize SS18-SSX/TLE1 co-localization in synovial sarcoma *in situ*, we set-up a proximity ligation assay (PLA) using antibodies specifically recognizing SS18 and TLE1. Nuclear proximity ligation signal is detectable between SS18 and TLE1 in all six tested synovial sarcoma patient derived cell lines: SYO-1 (SS18-SSX2), FUJI (SS18-SSX2), MoJo (SS18-SSX1) (Figure 1A); Yamato-SS (SS18-SSX1), and ASKA-SS (SS18-SSX1) (Supplementary Figure 1C); and CME-1 (SS18-SSX2) (Figure 2B). Weak or non-specific PLA signals are detectable in cells lines of other cancer subtypes (including three sarcomas bearing different fusion oncoproteins): MCF7 (breast carcinoma), HeLa (cervical carcinoma), A673 (Ewing sarcoma, EWS-FLI1) (Figure 1A), 402-91 (myxoid liposarcoma, FUS-DDIT3), SU-CCS-1 (clear cell sarcoma, EWS-ATF1), as well as single antibody, no antibody, and non-specific antibody conditions (Supplementary Figure 1A).

Quantification of SS18/TLE1 PLA signals in synovial sarcoma cell line nuclei is more than 10-fold higher than the level seen in control cell lines (Figures 1B and Supplementary Figure 1D). Co-immunoprecipitation analyses further demonstrate that the interaction of SS18-SSX with TLE1 is specific to synovial sarcoma, as SS18-SSX is pulled down with TLE1 exclusively in synovial sarcoma cell lines (Figure 1C). All cell lines used in this study express some level of SS18 and of TLE1; the lack of SS18 and TLE1 co-localization in SS18-SSX negative cell lines therefore indicates the nuclear proximity ligation signal is a result of the SS18-SSX/TLE1 interaction and not of wild-type SS18/TLE1 protein interactions (Supplementary Figure 1B).

### TLE1 co-localizes with SS18 only in the context of SS18-SSX

Reliable antibodies to detect SSX, suitable for co-IP or PLA assays, are currently not available. To determine whether SS18-SSX/TLE1 co-localization is specific for the fusion oncoprotein, knockdown of SS18-SSX was achieved with siRNA molecules (Figure 2A-2C) as well as shRNA vectors (Figure 2D-2F). When *SS18-SSX* expression is silenced, co-localization of SS18-SSX with TLE1 is lost and quantification of foci per nucleus is significantly decreased (Figure 2C, 2F). Both siRNA systems target *SSX* mRNA regions of the fusion transcript, and result in the specific silencing of SS18-SSX but not of endogenous SS18, bringing about a loss of SS18-SSX/TLE1 proximity ligation signals. This verifies previous results [5] which demonstrated that the interaction of SS18 with TLE1 occurs only in the context of the SS18-SSX fusion oncoprotein.

### Proximity ligation signals can be detected in FFPE synovial sarcoma tumor tissue samples

**Figure 3:**
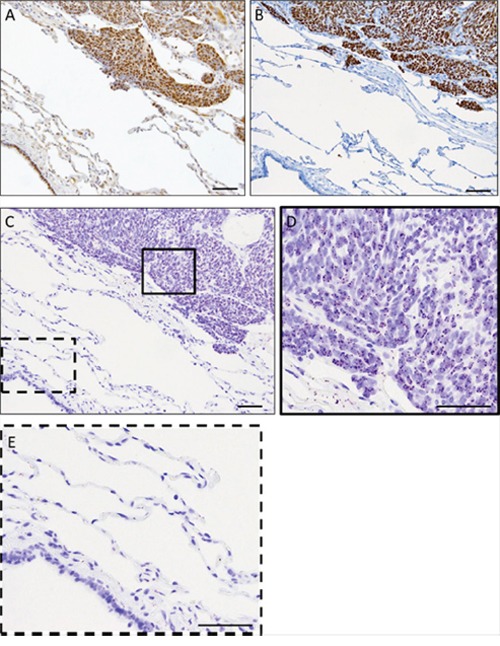
The PLA assay can be used to detect SS18-SSX/TLE1 co-localization in FFPE human synovial sarcoma tumor samples IHC staining of SS18 **A.** and TLE1 **B.** is strongly positive in synovial sarcoma tumor tissue from the metastasectomy specimen. The PLA nuclear signal is detected in the fixed human synovial sarcoma tumor tissue **C, D.** but not in the immediately adjacent normal lung tissue **C, E.** Scale bars represent 100 μm.

Formalin-fixed paraffin embedded (FFPE) patient-derived synovial sarcoma tumor samples were used to detect SS18-SSX/TLE1 co-localization in human tumor tissue samples. Immunohistochemical staining in synovial sarcoma patient surgical specimens demonstrated the presence of SS18-SSX and TLE1 as well as the specificity of TLE1 for synovial sarcoma cells (Figure 3A, 3B). In an excised pulmonary metastasis, SS18-SSX/TLE1 complex co-localization signal is detected exclusively in synovial sarcoma tissue nuclei (Figure 3C, 3D) while the adjacent normal lung tissues are negative (Figure 3E). The specificity of the proximity ligation signal in FFPE samples was additionally validated in embedded synovial sarcoma cell pellets in comparison to control sarcoma cell lines bearing different translocations (Supplementary Figure 2). SS18-SSX/TLE1 proximity ligation signal was detected only in synovial sarcoma samples.

### PLA enables *in situ* visualization of HDAC inhibitor-induced dissociation of SS18-SSX from TLE1

**Figure 4:**
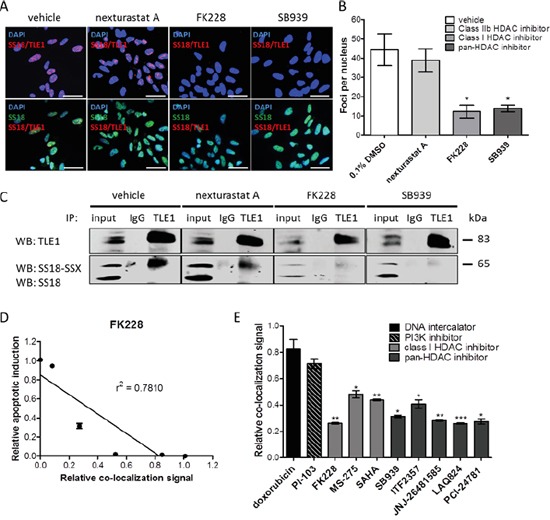
HDAC inhibitors disrupt SS18-SSX/TLE1 co-localization A significant decrease in detectable PLA signal following HDAC inhibition in SYO-1 cells **A, B.** is also confirmed by immunoprecipitation **C.** The decrease in PLA co-localization signal correlates with apoptosis induction by HDAC inhibitor FK228 in SYO-1 cells **D.** A panel of cytotoxic compounds and HDAC1/3 inhibitors were screened on SYO-1 cell at IC_50_ doses for 12 hours. While all compounds were effective in decreasing cell viability, only compounds inhibiting class I HDACs were found to decrease the PLA nuclear signal, as a result of disrupting SS18-SSX/TLE1 co-localization **E.** Scale bars represent 20 μm. Statistical significance compared to vehicle treatment controls was determined by Student t test: * denotes *p* < 0.05; ** denotes *p* < 0.01; *** denotes *p* < 0.001. Error bars represent standard error of mean from conditions performed in triplicate. Linear regression was measured by goodness of fit calculations in Prism Graphpad.

Previous studies have demonstrated that HDAC inhibitors disrupt the fusion oncoprotein complex in synovial sarcoma, re-establishing expression of tumor suppressor genes and apoptotic response [5, 7, 8]. To investigate if the SS18-SSX/TLE1 complex disruption induced by HDAC inhibitors can be detected *in situ* using the PLA method, SYO-1 synovial sarcoma cells were treated at IC_50_ doses with the class I HDAC inhibitors FK288 (romidepsin), MS275 (entinostat), SAHA (vorinostat) or the pan-HDAC inhibitor SB939 (pracinostat), as well as nexturastat A (a cytoplasmic class IIb HDAC6 inhibitor). SS18-SSX/TLE1 complex assembly following HDAC inhibition was assessed by proximity ligation signal in five synovial sarcoma cell lines (Figures 4A-4B and Supplementary Figure 3A-3F) and was confirmed by co-immunoprecipitation (Figure 4C).

Class I HDAC inhibitor-induced dissociation of SS18-SSX/TLE1 interactions is visualized as a significant decrease in PLA nuclear signal as well as a loss of SS18-SSX pulled down with TLE1 (Figure 4C). HDAC6 inhibition had minimal effect on SS18-SSX/TLE1 co-localization (Figure 4A-4C), supporting class I HDAC inhibition as being required for disruption of this complex. Dissociation of the SS18-SSX/TLE1 complex following HDAC inhibition correlates with apoptotic induction in synovial sarcoma cell lines (Figure 4D). The addition of proteasome inhibitor MG-132 does not prevent complex disruption, demonstrating that the observed dissociation of the SS18-SSX/TLE1 complex induced by class I HDAC inhibitors is not due to protein degradation (Supplementary Figure 3A-3B).

To demonstrate specificity of this drug-mediated protein complex dissociation, a panel of drug classes was tested concurrently in a 96-well high-throughput format, and the relative signal intensity of SS18-SSX/TLE1 co-localization was quantified (Figure 4E). SYO-1 cells were exposed to IC_50_ doses of the HDAC inhibitors FK228, MS-275, SAHA, SB939, ITF2357 (givinostat), JNJ-26481585 (quisinostat), LAQ824 (dacinostat), PCI-24781 (abexinostat), as well as doxorubicin (DNA intercalator) and PI-103 (PI3K inhibitor). Only class I HDAC inhibitors consistently disrupt SS18-SSX/TLE1 co-localization and decrease cell viability. By comparison, drugs primarily acting via DNA intercalation or PI3K inhibition significantly decrease cell viability without concomitant effects on the relative co-localization signal staining intensity of the fusion oncoprotein with its partner.

### PLA detection of oncoprotein interactions can be used in high-throughput drug screens to identify molecules that disrupt the SS18-SSX oncoprotein complex

**Figure 5:**
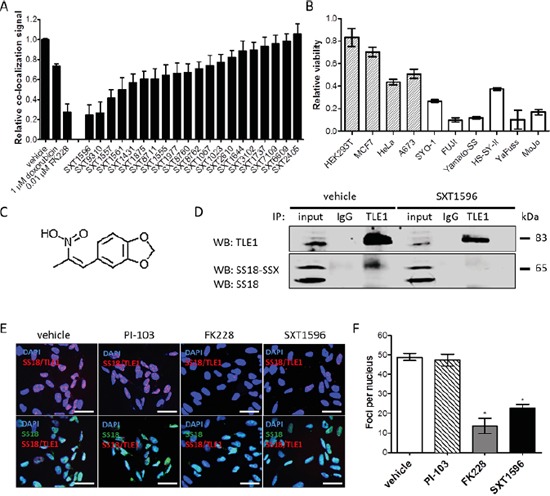
The PLA assay can be used for high-throughput drug screening for targeted compounds in synovial sarcoma From a 16 000 compound small molecule drug library, a compound designated SXT1596 was recognized to significantly decrease relative co-localization of SS18-SSX/TLE1 in human synovial sarcoma cells **A.** SXT1596 selectively decreases cell viability in synovial sarcoma cell lines, SYO-1, FUJI, Yamato-SS, HS-SY-II, YaFuss and MoJo (white bars), when compared to negative control cell lines HEK293T, MCF7, HeLa and A673 (shaded bars) at a concentration of 5 μM **B.** Negative control cell lines HeLa and A673 are sensitive to SXT1596 at higher doses. SXT1596 (5-(2-Nitro-1-propenyl)-1,3-benzodioxole) (structure shown in **C.**) mediated disruption of the SS18-SSX/TLE1 association was confirmed by immunoprecipitation **D.** Nuclear SS18-SSX/TLE1 PLA signal in SYO-1 cells decreased following SXT1596 treatment to a degree similar to that obtained with the HDAC inhibitor FK228 **E, F.** Statistical significance compared to vehicle treatment controls was determined by Student t test: * denotes *p* < 0.05. Error bars represent standard error of mean from conditions performed in triplicate. Small molecule **C.** was drawn using iChem Labs ChemDoodle software. Scale bars represent 20 μm.

A high-throughput drug screen consisting of 16 000 small molecule inhibitors (Maybridge Chemicals) was completed in synovial sarcoma cell lines expressing the SS18-SSX oncoprotein. Compounds eliciting a decrease in cell viability of greater than 50% were assessed for disruptions to SS18-SSX/TLE1 co-localization in a high-throughput 96-well PLA format. At IC_50_ doses, the top 20 hits from the viability assessment had different degrees of concurrent effect on the protein co-localization measures (Figure 5A). The top three available compounds capable of disrupting the SS18-SSX/TLE1 proximity ligation signal were validated in multiple synovial sarcoma cell lines. One compound (designated SXT1596) demonstrated particular efficacy in decreasing cell viability selectively in synovial sarcoma cell lines (Figure 5B-5C). Compound SXT1596 effectively disrupts the interaction between SS18-SSX and TLE1, as confirmed by PLA and immunoprecipitation (Figure 5D-5F).

### Novel compound SXT1596 re-establishes normal cell signalling in synovial sarcoma

**Figure 6:**
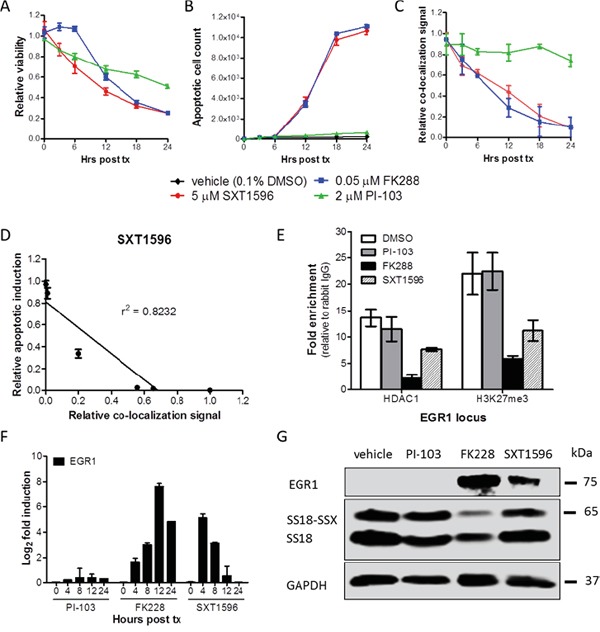
SXT1596 decreases cell viability and reactivates EGR1 expression in synovial sarcoma The small molecule SXT1596 induces apoptosis at a similar rate to that of HDAC inhibitor FK228 in SYO-1 cells **A, B.** and brings about a consistent decrease in SS18-SSX/TLE1 PLA co-localization signal in SYO-1 cells **C.** The induction of apoptosis correlates with the decrease in PLA co-localization signal **D.** Chromatin immunoprecipitation at the *EGR1* promoter reveals a decrease in H3K27me3 enrichment following SXT1596 treatment **E.** SXT1596 reactivates *EGR1* expression, at the RNA **F.** and protein **G.** level. Error bars represent standard error of mean from conditions performed in triplicate. Linear regression was measured by goodness of fit calculations in Prism Graphpad.

Treatment of SYO-1 cells with SXT1596 resulted in significantly decreased cell viability (Figure 6A) and increased apoptosis (Figure 6B) that correlated with the decrease in SS18-SSX/TLE1 co-localization signal (Figure 6C-6D). Chromatin immunoprecipitation revealed a decrease in HDAC1 and H3K23me3 at the *EGR1* promoter, a known target for repression by the SS18-SSX/TLE1/ATF2 complex consistent with a disruption of the biologic effects on SS18-SSX target genes in synovial sarcoma (Figure 6E) [5, 7, 8]. Similar to class I HDAC inhibitors, the compound SXT1596 reactivates EGR1 transcription and protein expression (Figure 6F-6G). SS18-SSX protein levels remain relatively constant following SXT1596 treatment, whereas the FK228 HDAC inhibitor leads to oncoprotein degradation (Figure 6G).

## DISCUSSION

In this study, the proximity ligation assay was applied to a neoplasm driven by a fusion oncoprotein, in this case confirming the key SS18-SSX interaction with TLE1 in synovial sarcoma cells and patient samples. This method can assess the capacity for drugs to disrupt the relevant functional interactions of a mutant oncoprotein, and can be used in high-throughput drug screens. As t(X;18) is the sole driving cytogenic event in synovial sarcoma, inhibiting the interactions of the resulting SS18-SSX chimeric oncoprotein with its effector partners can prevent aberrant transcriptional repression, potentially reverse oncogenic effects and initiate apoptosis. PLA allows for quantification of the loss of protein interactions, which can be used to assess drug action and select the most potent agents in this model.

In a high-throughput PLA format, inhibitors capable of disrupting the driving complex can be revealed quickly, *in situ*. This is an efficient means to screen large compound libraries for their capacity to disrupt the relevant oncogenic interactions. In addition, in contrast to fluorescence resonance energy transfer (FRET) methodology in which fluorescent probes must be engineered onto proteins of interest [21], PLA requires no synthetic protein modifications (that may obstruct normal protein complex interactions). Physical interactions among proteins are time and cell context dependent, and the detection of a particular protein-protein interaction in a generic experimental condition or in cell culture models may not accurately represent the situation in patients.

PLA is performed in the context of the cell's naturally occurring biology, reducing the likelihood of artifactual alterations to SS18-SSX interactions (which are likely to occur, for example, when SS18-SSX is forcibly overexpressed in vector-based systems as opposed to being expressed under its endogenous promoter at physiological levels in its original cellular background). This methodology therefore anticipates a streamlined approach to screen for effective therapeutics targeting the driving complex in synovial sarcoma, and will be similarly applicable in other cancers driven by fusion transcription factors. These include other sarcomas and hematopoietic neoplasms typically afflicting young patients, as well as an increasing number of other tumors wherein recurrent fusions are being identified by RNA-Seq technologies [22].

The compound designated SXT1596 (5-(2-Nitro-1-propenyl)-1,3-benzodioxole) identified in the PLA drug screen to decrease SS18-SSX/TLE1 co-localization is a biologically active chemical. A number of similar 1,3-benzodioxole derivatives have demonstrated anti-tumor activity by various mechanisms, including binding to tubulin and heat-shock protein inhibition [23-25]. In addition, the trans-β-Nitrostyrene domain possessed by SXT1596 has been observed in related compounds to inhibit protein phosphatases [26] and telomerase [27], resulting in anti-tumor action in several cancer subtypes [28-30]. Whether the compound directly inhibits the SS18-SSX/TLE1 interaction domain or disrupts the complex by a secondary mechanism is currently unknown, but the structure of SXT1596 indicates that it is likely a covalent protein inhibitor, not expected to be well tolerated until further pharmacologic optimization steps are carried out [31].

Driving protein complexes are often recruited and deregulated in translocation-associated sarcomas [32]. Uncovering new methods of study and utilizing emerging technologies such as the proximity ligation assay may allow better means to identify therapeutic strategies. In synovial sarcoma, this technique can be used in a high-throughput format to screen targeting compounds as a means of identifying novel agents with disease-specific activity. This methodology should also be applicable in other types of translocation-associated cancers.

## MATERIALS AND METHODS

### Cell culture and chemicals

Six human synovial sarcoma cell lines were kindly provided: SYO-1 (Dr. Akira Kawai, National Cancer Centre Hospital, Tokyo, Japan), FUJI (Dr. Kazuo Nagashima, Hokkaido University School of Medicine, Sapporo, Japan), YaFuss, HS-SY-II (Dr. Scott Lowe, Memorial Sloan Kettering Cancer Centre, New York, USA), MoJo (Dr. K. Jones, University of Utah, Salt Lake City, UT) CME-1 (Dr. J.C. Knight, UMDS, London), Yamato-SS and ASKA-SS (Dr. K. Itoh, Osaka Medical Center for Cancer and Cardiovascular Diseases, Japan). For use as controls, A673 (Ewing sarcoma) and SU-CCS-1 (clear cell sarcoma) lines were purchased from the ATCC, while 402-91 (myxoid liposarcoma) was received from Dr. Pierre Aman, University of Gothenburg, Sweden. Sarcoma cell lines were maintained in RPMI-1640 medium with 10% fetal bovine serum (FBS) (Life Technologies). As additional human non-sarcoma controls, breast cancer cell line MCF7 and cervical cancer cell line HeLa were purchased from the ATCC and cultured in DMEM medium with 10% FBS. All cells were grown at 37°C, 95% humidity, and 5% CO_2_.

Pharmacologic compounds were purchased from Selleck Chemicals (Houston, TX, USA). Small molecule inhibitor library compounds were purchased from Maybridge Chemicals (Waltham, MA, USA).

### Tissue sections

Paraffin embedded tissue from a synovial sarcoma lung metastasis was obtained from the Vancouver General Hospital. Synovial sarcoma and control cell lines were fixed overnight in 10% NBF and embedded in paraffin as previously described [33]. FFPE tissue and embedded cell line pellets were sectioned at 4 μm onto micro-slides for use in both conventional immunohistochemistry and proximity ligation assay procedures. Immunohistochemistry staining was carried out using the Ventana automated staining platform, with primary antibody at a 1/150 dilution for both SS18 (Santa-Cruz Biotechnologies, sc-28698) and TLE1 (Origene, TA800301).

### Proximity ligation assay

Cells were seeded in culture treated chamber slides or in 96 well plates. The following day, wells were treated with pharmacologic agents for 0, 4, 8, or 12 hours. Cells were washed twice with PBS, fixed with 3% formaldehyde and permeabilized with 0.1% Triton X-100. Wells were blocked with blocking buffer and incubated overnight at 4°C with primary antibodies at a 1/1000 dilution: SS18 (rabbit polyclonal, Santa-Cruz Biotechnologies, sc-28698) or TLE1 (mouse monoclonal, Origene, TA800301). Proximity ligation was performed utilizing the Duolink^®^ In Situ Red Starter Kit Mouse/Rabbit (Sigma-Aldrich) according to the manufacturer's protocol. The oligonucleotides and antibody-nucleic acid conjugates used were those provided in the Sigma-Aldrich PLA kit (DUO92101). Alexa Fluor 488 secondary antibody (Life Technologies) was added during signal amplification to confirm the presence of nuclear proteins. Fluorescence was detected using a Zeiss Axioplan2 microscope at 40x. Images were quantified in triplicate using ImageJ software (NIH) as foci per nucleus, defined as the number of interaction points counted per nucleus. Co-localization fluorescence signal staining intensity was detected using the automated Cellomics ArrayScan VTI compartmentalization analysis software (Thermo Scientific). Twenty images per well were quantified. Relative PLA stain signal was normalized to the vehicle (0.1% DMSO) treated control as well as to TLE1 antibody-only control, to account for background signal.

Proximity ligation in FFPE samples was performed according to the manufacturer's brightfield protocol. Sections were heated at 60°C for one hour, then de-paraffinized in Citrisolv (Fisher Scientific) and rehydrated using an ethanol gradient. Antigen retrieval was performed with Borg Decloaker (Biocare) in a water bath heated at 95°C for 20 minutes. The slides were cooled to room temperature in antigen retrieval solution for 10 minutes and in a cold water bath for an additional 10 minutes. Endogenous peroxidase activity was blocked with 2% hydrogen peroxide for 10 minutes at room temperature, and cell membranes permeabilized with 0.1% Triton X-100 in PBS for 5 minutes. Slides were incubated with Dako protein block for 30 minutes at room temperature to eliminate nonspecific protein background. The antibody pair was diluted using Dako antibody diluent and incubated on the slides overnight. The proximity ligation procedure was carried out the following day. Images were captured using an Olympus BX46 brightfield microscope with a WHN10x-h/22 eyepiece and an Olympus DP21 camera.

### Cell viability assays

Cells were seeded in 96 well plates and treated in triplicate at IC_50_ doses of the tested pharmacological agents. IC_50_ doses were determined in synovial sarcoma cell lines by dose curve studies. Cell viability was assessed as compared with the vehicle 0.1% DMSO condition at 48 hours post treatment using MTS reagent (Promega). Cell confluency and apoptosis induction was assessed over a 48-hour timeframe utilizing the IncuCyte Zoom^®^ live cell imaging software (Essen BioScience). Apoptosis was assessed by IncuCyte™ Kinetic Caspase-3/7 Apoptosis Assay Reagent (Essen BioScience, 4440).

### Co-immunoprecipitation and western blots

According to the manufacturers protocol, 6 μg of antibody (TLE1: Abcam, ab125183; normal rabbit IgG: Santa-Cruz Biotechnologies, sc-2027) in PBST was added to 50 μL of Dynabeads^®^ Protein G magnetic beads (Life Technologies) for 30 minutes with rotation at room temperature. Antibody was then crosslinked to the beads with the addition of 5 mM BS^3^ (ThermoScientific, 21580) in conjugation buffer for 30 minutes with rotation at room temperature. Crosslinking was quenched with Tris-HCl pH 7.5 for 15 minutes at room temperature. Beads were then washed 3 times with conjugation buffer and once with PBST. Following drug treatment, cells were washed with ice-cold PBS and incubated with Triton X-100 (Sigma-Aldrich) lysis buffer for 30 min on ice, with inversion every 10 minutes. Whole cell lysates were centrifuged at 4°C, 10 000 rpm for 15 minutes. Supernatants were quantified and 1000 μg of protein was mixed with the antibody-crosslinked beads. The immunoprecipitation was incubated overnight at 4°C with rotation. The supernatant was then removed and the beads were washed 3 times in lysis buffer and 3 times in PBST, and then boiled in 2X loading dye for 5 minutes.

Samples were separated by 10% SDS-PAGE and transferred to PVDF membranes (Bio-Rad Laboratories). Blots were incubated with indicated antibodies (TLE: Origene TA800301; SS18: Santa Cruz Biotechnologies, sc-28698; EGR1: Cell Signalling, 4153S; GAPDH: Santa Cruz Biotechnologies sc-25778). Signals were visualized using the Odyssey Infrared System (LI-COR Biosciences).

### RNA interference

Duplex oligo (sense, CAAGAAGCCAGCA GAGGAATT; antisense, UUCCUCUGCUGGCUUCU UGTT) *SS18-SSX2* siRNAs were designed to target the *SSX* portion of *SS18-SSX* using the Integrated DNA Technologies RNA interference (RNAi) design tool, and synthesized by Integrated DNA Technologies (IDT) as previously described [7]. SYO-1 cells were seeded in 6 well plates. At 60% confluence, cells were transfected with 30-50 pmol si*SS18-SSX2* and 9 μL Lipofectamine RNAiMax transfection reagent (Invitrogen) in Opti-MEM serum free media (Life Technologies), according to the manufacturer's protocol. Protein was harvested 48 hours post transfection, and knock-down confirmed by western blot with SS18 antibody (Santa Cruz Biotechnologies).

The synovial sarcoma CME-1 cell line was stably transfected with a doxycycline-inducible shRNA targeting *SS18-SSX*. CME-1 cells were used for these experiments because they are more easily transfected than other available synovial sarcoma cell lines. The inducible line was generated with the use of a pcDNA6 TR and subsequently with the pSuperior shRNA vector encoding a target oligonucleotide specific for the 3′ UTR region of *SS18-SSX* (1, 2 and 4), as previously described [34, 35]. shRNA-mediated knockdown of SS18-SSX was induced by the addition of doxycycline (Clontech). Protein was harvested 24 hours post transfection and knock-down was confirmed by western blot with SS18 antibody (Santa Cruz Biotechnologies).

### High-throughput drug screen assay

The 16 000 compound Maybridge Chemicals library drug screen was undertaken in SYO-1 cells. Cells were seeded in 96 well plates at 10 000 cells/well. The following day, compounds from the Maybridge library were transferred from stock plates (5 mM in DMSO) using a Biorobotics Biogrid II pinning robot with a 96-pin tool diameter of 0.4-mm, to bring about a final concentration of approximately 5 μM per well. Plates were developed with MTS reagent 24 hours post treatment to select for direct effects at this dose. Hits bringing about a decrease in viability of greater than 50% were validated in a dose-response curve and in additional synovial sarcoma cell lines. IC_50_ doses of the small molecules were used for PLA validation studies.

### Chromatin immunoprecipitation (ChIP)

ChIP experiments were performed following the Active Motif ChIP-IT Express Enzymatic kit protocol. Cells were cross-linked with 1% formaldehyde prior to lysis and homogenization. Cross-linked DNA was sheared by enzymatic digestion. After centrifugation, the supernatants were incubated with the indicated antibody at 4°C overnight. Precipitates were washed, eluted with 1% SDS and incubated at 65°C to reverse crosslinking. ChIP-enriched DNA was purified by phenol-chloroform (Invitrogen) and ethanol precipitation, and was subjected to SYBR Green qPCR analysis (Roche) using an ABI ViiA7 qPCR system and an *EGR1* promoter primer set (hEGR1 -0.1 kb/CRE sense: TAGGGTGCAGGATGGAGGT, antisense: AAGCAGGAAGCCCTAATATGGCAG) [5].

### Real-time qPCR

Total RNA was isolated from treated cells using the RNeasy Mini kit (Qiagen) and was then reverse transcribed to cDNA using Oligo(dT) (Invitrogen) and Superscript III (Invitrogen). SYBR Green (Roche) reagent was used for qPCR expression analysis, using an ABI ViiA7 qPCR system. The following primers for *EGR1* expression were used; sense: AGCCCTACGAGCACCTG, antisense: CGGTGGGTTGGTCATG. All transcript levels were normalized to *GAPDH* RNA expression.

## SUPPLEMENTARY FIGURES


