# Supplementary material for: Identification of cytotoxic agents disrupting synovial sarcoma oncoprotein interactions by proximity ligation assay
Source: Oncotarget. 2016 Apr 21;7(23):34384–94. doi: 10.18632/oncotarget.8882 (PMC5085163; doi:10.18632/oncotarget.8882)
Supplement: Supplementary file 1 [file oncotarget-07-34384-s001.pdf]

Identification of cytotoxic agents disrupting synovial sarcoma oncoprotein interactions by proximity ligation assay

SUPPLEMENTARY FIGURES

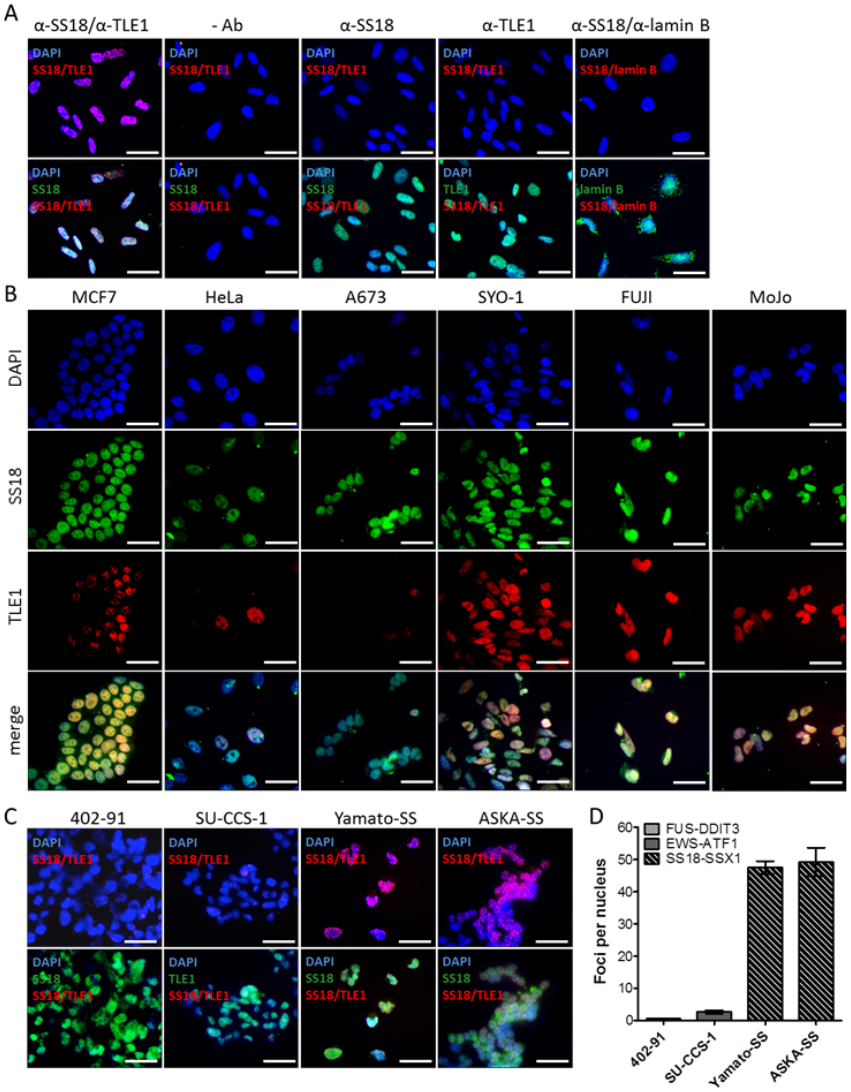

Supplementary Figure S1: The proximity ligation assay (PLA) demonstrates SS18-SSX/TLE1 co-localization selectively when SS18 (rabbit) and TLE1 (mouse) antibodies are included. Control experiments with no antibody, single antibody or non-specific antibody inclusion resulted in little to no nuclear signal in SYO-1 cells **A.** All tested cell lines express some level of detectable SS18 (green channel) and TLE1 (red channel), but only SS18-SSX-containing cell lines stain positive for nuclear SS18-SSX/TLE1 PLA signals **B.** Beyond the cell lines that could be shown in Figure 1 of the main paper, additional SS18-SSX-containing synovial sarcoma cell lines Yamato-SS and ASKA-SS were also positive for SS18-SSX/TLE1 co-localization, whereas additional translocation-associated sarcoma cell lines (402-91 myxoid liposarcoma, SU-CCS-1 clear cell sarcoma) were not **C, D.** Scale bars represent 20 µm. Error bars represent standard error of mean from three images.

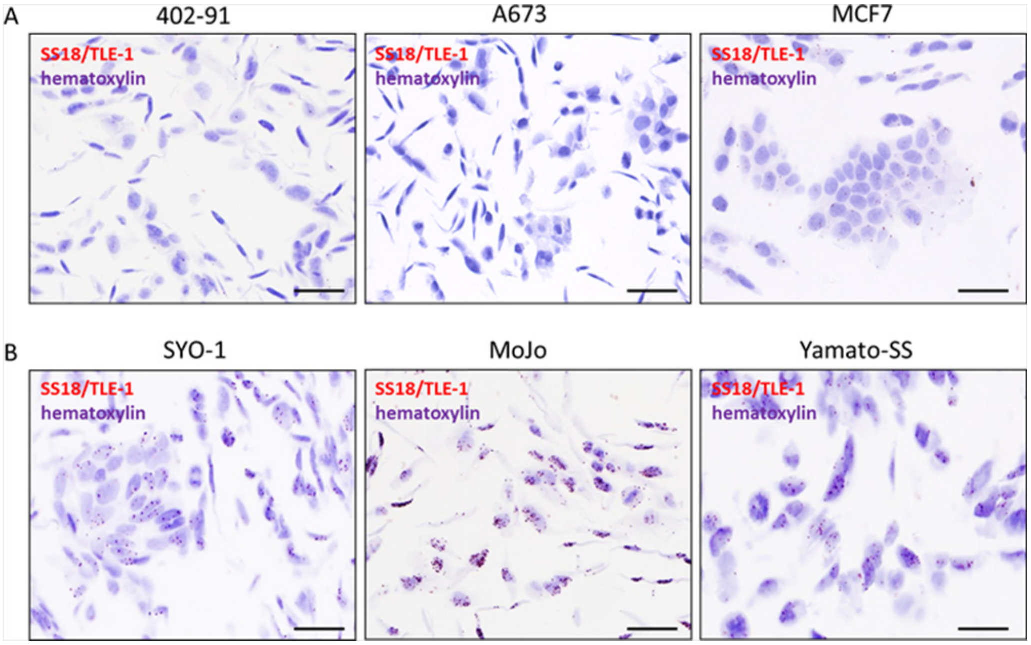

Supplementary Figure S2: The PLA assay can detect SS18-SSX/TLE1 co-localization in formalin fixed paraffin embedded (FFPE) pelleted cell samples. Nuclear PLA signal is selectively detected in synovial sarcoma cell lines **B.** and not in cell lines from other types of cancer: 402-91 myxoid liposarcoma, A673 Ewing sarcoma, MCF7 breast cancer **A.** Scale bars represent 20 µm.

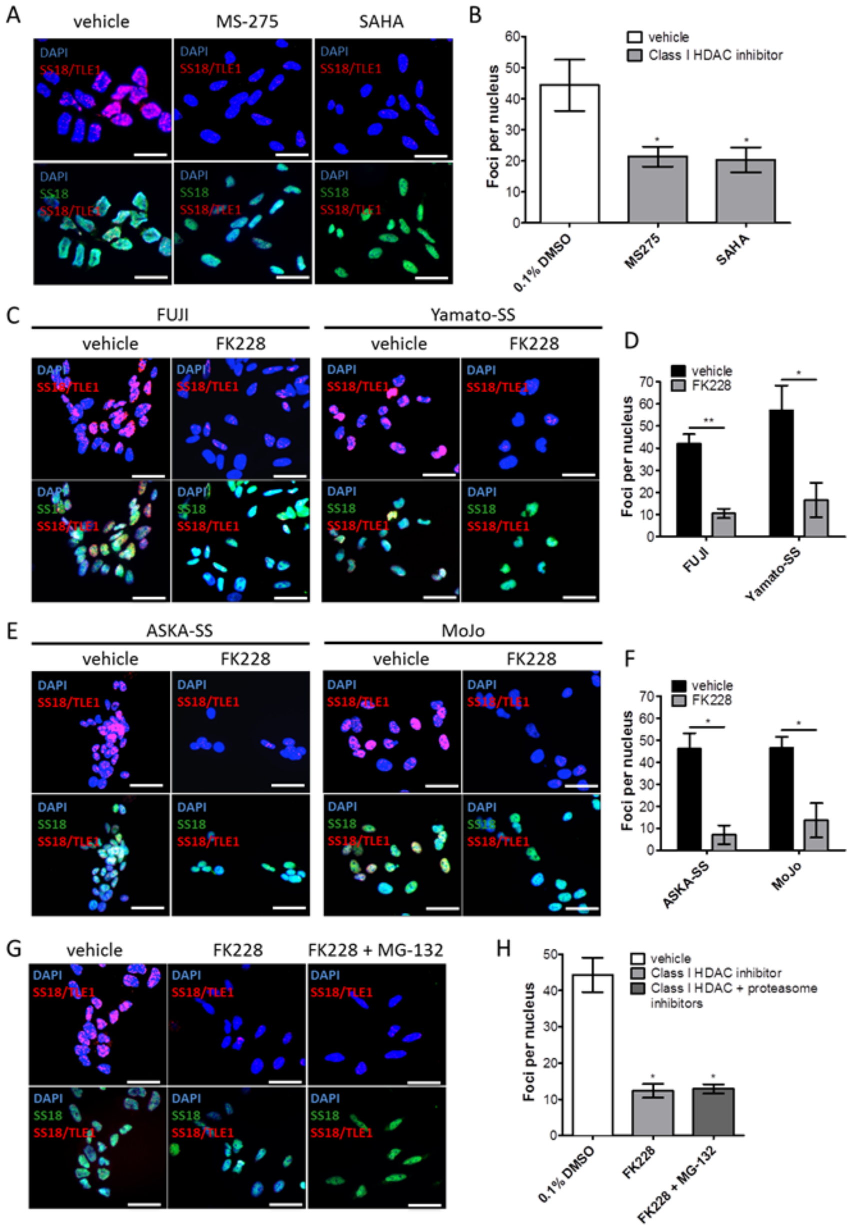

Supplementary Figure S3: Class I HDAC inhibitors significantly decrease nuclear PLA signal for SS18-SSX/TLE1 in synovial sarcoma. At 12 hours post treatment in SYO-1 cells, nuclear PLA signal is decreased following incubation with IC_{50} doses of MS-275 and SAHA **A, B.** Class I HDAC inhibition consistently brings about a significant decrease in PLA signal in four additional human synovial sarcoma cell lines: FUJI, Yamato-SS, ASKA-SS, and MoJo **C-F.** Class I HDAC inhibition dissociates SS18 from TLE1, consistently with and without the addition of a proteasome inhibitor, indicating loss of PLA signal is not solely due to a loss of SS18-SSX protein **G, H.** Scale bars represent 20 µm. Statistical significance compared to vehicle treated controls was determined by Student t test: * denotes $p < 0.05$; * denotes $p < 0.05$; ** denotes $p < 0.01$. Error bars represent standard error of mean from three images.
